# Is Urban Household Emergency Preparedness Associated with Short-Term Impact Reduction after a Super Typhoon in Subtropical City?

**DOI:** 10.3390/ijerph16040596

**Published:** 2019-02-19

**Authors:** Emily Ying Yang Chan, Asta Yi Tao Man, Holly Ching Yu Lam, Gloria Kwong Wai Chan, Brian J. Hall, Kevin Kei Ching Hung

**Affiliations:** 1 Collaborating Centre for Oxford University and CUHK for Disaster and Medical Humanitarian Response (CCOUC), The Chinese University of Hong Kong, Hong Kong, China; asta.man@cuhk.edu.hk (A.Y.T.M.); hollylam@cuhk.edu.hk (H.C.Y.L.); gloria.chan@cuhk.edu.hk (G.K.W.C.); 2 Nuffield Department of Medicine, University of Oxford, Oxford OX37BN, UK; 3 Global and Community Mental Health Research Group, Faculty of Social Sciences, University of Macau, Macao, China; brianhall@um.edu.mo; 4 Accident & Emergency Academic Unit, The Chinese University of Hong Kong, Prince of Wales Hospital, Hong Kong, China; kevin.hung@cuhk.edu.hk

**Keywords:** typhoon, hurricane, cyclone, strong wind levels, natural disaster, Health-EDRM, household preparedness, urban, climate change related extreme events, subtropical city

## Abstract

Climate change-related extreme events are increasing in frequency and severity. Understanding household emergency preparedness capacity in Health-Emergency and Disaster Risk Management (Health-EDRM) for at risk urban communities is limited. The main objective of the study is to explore the association among risk perception, household preparedness, and the self-reported short-term impacts of Typhoons for urban residents. A population-based, cross-sectional telephone survey using random digit-dialling was conducted among Hong Kong adults within 2 weeks following 2018 Typhoon Mangkhut, the most intense typhoon that affected Hong Kong, a subtropical city, in thirty years. Among the 521 respondents, 93.9% and 74.3% reported some form of emergency preparedness and typhoon-specific preparedness measure (TSPM) against Mangkhut, respectively. Respondents who perceived a higher risk at home during typhoons and had practiced routine emergency preparedness measures (during nonemergency periods) were more likely to undertake TSPM. Of the respondents, 33.4% reported some form of impact (11.1% were household-specific) by Typhoon Mangkhut. Practicing TSPM was not associated with the reduction of short-term household impacts. Current preparedness measures may be insufficient to address the impact of super typhoons. Strategies for health-EDRM for urban residents will be needed to cope with increasing climate change-related extreme events.

## 1. Introduction

Typhoons, also known as cyclones or hurricanes depending on its location and strength, are the most common natural hazard in the Asia Pacific and Southeast Asia region [1]. Doocy et al. highlighted although the global hurricane/typhoon-related mortality trends have decreased in the past seventy years, severe typhoons have increased in frequency in the last decade. Hong Kong, a densely populated subtropical metropolis in Southern China, has on average 5–6 annual typhoons [2]. Between 1980–2010, only two T10 typhoons, the highest Tropical Cyclone Warning Signal, occurred, yet three happened in one decade (2012, 2017, and 2018) [2]. Since 2018, the last typhoon-related death was recorded in 1999; between 129 and 458 people were injured in the last three T10 typhoons. These climate-related events have brought on landslides, torrential rain, and flooding which led to cascading impacts on the city. With climate change, more severe typhoons are expected, and the coping capacity of typhoon-prone densely populated urban cities needs to be explored in order to plan for effective disaster risk reduction strategies.

Health-emergency and disaster risk management (Health-EDRM) encompasses the systematic analysis and management of health risks through the reduction and mitigation of hazards and vulnerability in all stages of the disaster management cycle. Preventive measures to mitigate disaster-related health risks are needed, which includes assessing the individual, household, and community’s capacity for food security, clean water and sanitation, and injury prevention [3,4,5]. Health-EDRM research in Asia suggests that despite high knowledge about typhoons, people have a low self-perception of the associated health risks and many do not adhere to government warnings [6,7,8].

Recent studies in Hong Kong, a subtropical city, for urban disaster and emergency preparedness found just over 75% of residents had some form of household emergency preparedness items (first-aid kit, basic aid supplies, emergency food and drinking water, basic medication, and/or a fire extinguishing equipment) [9]. Other studies in the same city indicated 69% of residents had not taken any precautions when a severe weather warning was announced [10], and in a subsequent study, 82.3% did not perceive Hong Kong to be a city susceptible to disasters [11]. Few relayed the correct responses to a typhoon warning signal, and they did not have adequate first-aid knowledge [12]. Notably, how personal risk perceptions and routine emergency preparedness might be associated with household preparedness in extreme meteorological events have yet to be studied in Asia.

Typhoon Mangkhut, which swept across the Philippines, Hong Kong, and Macao in September 2018, was the second most intense storm which battered Hong Kong since recording began in 1946 and was the most intense storm in the past three decades [13]. The typhoon, which landed in September, had wind speeds reaching 161 km/h in Hong Kong and was classified as a T10; only sixteen of such Tropical Cyclone Warning Signals have been hoisted in over seventy years [2,14]. It is equivalent to a category two Hurricane according to the Saffir–Simpson Hurricane Wind Scale, comparable to the 2018 Hurricane Katia which left parts of Mexico in disarray [15]. The Philippines reported over 100 deaths; while in Hong Kong, there were no fatalities, over 450 people were injured [16,17]. The objectives of this paper are (1) to examine the household preparedness measures conducted, the responsive activities undertaken, and the short-term impacts experienced by the Hong Kong community during Typhoon Mangkhut; (2) to identify the associating sociodemographic factors of preparedness, response, and impact; and (3) to explore the associations between risk perceptions, preparedness, responses, impacts, and attitudes for future preparation. The findings will bridge the current knowledge gaps associated with urban Health-EDRM for climate change related extreme events in Asia.

## 2. Materials and Methods

A cross-sectional study, population-based, household telephone survey using random digit dialling, was conducted just over two weeks after Typhoon Mangkhut’s landing in September 2018. The “last birthday method” was used to ensure randomization at the household level. The interviewer would seek the household member whose birthday was the closest to the interview date to ensure the respondent was randomly selected within the family unit. Hong Kong residents who understood Cantonese and were 18 years old or above were interviewed. Verbal informed consent was obtained at the start of each interview, and ethics approval was sought from the Survey and Behavioural Research Committee at The Chinese University of Hong Kong (SBRE-18-075).

A multiple choice-based survey was used to collect self-reported information: (a) sociodemographic information, (b) the floor lived on, (c) risk perception toward typhoons, (d) routine household preparedness, (e) household preparedness measures for Typhoon Mangkhut, (f) activities conducted during the typhoon period (whether they had left home when a typhoon signal T8 or higher was hoisted, (g) whether they had paid attention to the weather conditions and the source of the information, and (h) the short-term impact brought on by Mangkhut. Other impacts reported but not included in the multiple-choice options were recorded separately in text format. Short-term impact refers to any impact occurring from the time when Typhoon Mangkhut approached Hong Kong to the date of interview (from immediate to two weeks after landfall). Respondents were also asked to comment on the information provided by the Hong Kong Government regarding Typhoon Mangkhut and their willingness to prepare for future typhoons.

For the routine household preparedness measures, general preparedness actions enquired included if basic supplies may be available for injury and wound management, possession of medication to manage existing health conditions, and any other measures to maintain a general state of health (Table 1). For the household preparedness measures for Typhoon Mangkhut, it included the combination of the routine measures mentioned above and the typhoon-specific preparedness measures (TSPM). Specifically, TSPM included (1) retrieving/storage of outdoor items that could be blown away, (2) applying anti-leaking or anti-seeping measures, and (3) taping windows (Table 1).

Statistical analyses were performed using IBM SPSS, version 24. Descriptive chi-square (or *X^2^*) tests and multivariable logistic regression were performed to identify the associations among typhoon preparedness, typhoon risk perception, and sociodemographic factors. Covariates were selected for the multivariable model based on the chi-square tests (*p* < 0.10). All odds ratios (OR) present in this paper were adjusted odds ratios from the multivariable models.

## 3. Results

Data collection was completed within 16 days (17 September 2018 to 2 October 2018) after the landfall on 16 September 2018. The final sample size constituted to 521 valid respondents (response rate was 31.6% among valid telephone numbers called), and the study sample comprised of 57.6% women, 41.7% aged below 45, just over 50.0% that attained a post-secondary education, and 42.0% that had a monthly household income of over $40,000 HKD (Table 2). The study sample was comparable to the 2016 Hong Kong Census for gender and residential district, and consisted of slightly more middle-aged adults (Table 2).

### 3.1. Risk Perception and Preparedness Activities

Of the 521 respondents, 9.4% perceived their home to be at high risk of danger during typhoons. For preparedness efforts during nonemergency periods, the most commonly reported routine preparedness measures included the procession of basic first-aid supplies, e.g., Band-Aid’s and ace bandages (95.2%); basic medication, e.g., pain killers (93.7%); and food supply (82.9%) (Table 1). Specifically for Typhoon Mangkhut, the three most frequently reported routine preparedness measures undertaken (out of six) included having extra food supplies (68.1%), a backup light source (54%), and having basic medication (46.4%). When examining TSPM for Typhoon Mangkhut, 74.3% of respondents practiced at least one (i.e., retrieved/stored outdoor items that could be blown away, applied anti-leaking or anti-seeping measures, or taped windows). Of note, 6.1% of the respondents reported undertaking no preparedness measure at all for Typhoon Mangkhut.

No significant associations were found between sociodemographic factors and a high typhoon risk perception (*p* > 0.20) both before and after adjusting for the height of the residential location. Results of univariable analyses (Table 3) showed a significant association between practicing at least one TSPM and education (*p* < 0.001), age group (*p* < 0.001), and perceived risk (*p* = 0.023). The association was sustained in the multivariable analyses for education (secondary vs. post-secondary, OR = 0.48; 95% CI = 0.03–0.77), age group (25–44 vs. 65+, OR = 2.80; 95% CI = 1.39–5.65), and high typhoon risk perception (vs. low perception, OR = 2.63; 95% CI = 1.07–650). Routine household preparedness was also found to be associated with practicing at least one TSPM. Of those nine routine measures, four measures were significantly associated with practicing at least one TSPM after adjusting for gender, age, education, and risk perception. They were having had a first-aid kit (OR = 1.79; 95% CI = 1.18–2.73), food supply (OR = 1.80; 95% CI = 1.08–3.03), fire extinguishing equipment (OR = 3.13; 95% CI = 1.36–7.20), and a backup light source (OR = 1.69; 95% CI = 1.03–2.77) (Table A1).

### 3.2. Responsive Activities Undertaken during the Typhoon

The three main routes of seeking weather-related information about Typhoon Mangkhut was television (52.6%), mobile apps (24.6%), and internet-based websites (14.2%). With the exception of website usage, which had no special association with gender, women were more likely to use any of the information channels mentioned above than men (*p* = 0.002). Most respondents (87.9%) felt the Hong Kong Government provided enough information for respondents to prepare and respond to Typhoon Mangkhut. Of public safety concern, 16.0% of respondents reported leaving their homes when the storm was at its height of strength (i.e. the warning signal was T8 or above). Among those who left their home, 74.7% reported such behaviour was for non-occupational and nonemergency related reasons (such as going out for meals and to cinemas). Adjusted for gender and age, men (OR = 1.98; 95% CI = 1.22–3.22) and younger groups (18–24 vs. ≥65; OR = 3.40; 95% CI = 1.29–8.93) were found more likely to go out for nonemergency and non-occupational purposes when the warning signal was T8 or above.

### 3.3. Impact of the Typhoon

Of all the respondents, 33.4% reported experiencing short-term impacts resulted from the typhoon. The most common impacts included road or traffic blockages which prevented respondents from going to work or school after landfall (70.7%), power outages (14.9%), and home damages during the typhoon (13.8%); less than 6% reported financial loss (of any form, such as cancelled trips and business losses), item loss, and injury. Specifically for household impacts, 11.1% reported being affected, which cited household damages, the loss of property, and power outages as the most common impacts. Our study sample showed 4 (0.77%) respondents reported injuries, which were related to slipping and wounds from broken glass.

Among the 74.3% who had practiced at least one TSPM for Typhoon Mangkhut, 37.2% reported short-term impacts (12.9% specific to household impacts) by the typhoon. The univariable analysis found respondents who did at least one TSPM were associated with a higher risk of having a household impact due to Typhoon Mangkhut (*p* = 0.027) (Table 4). This association, however, became statistically nonsignificant after adjusting for the perceived risk of typhoons.

### 3.4. Experiences and Future Preparedness

Half of respondents (49.9%) indicated they would prepare for future typhoons. Multivariable analysis indicated that respondents who had applied at least one TSPM against Typhoon Mangkhut (OR = 3.07; 95% CI = 1.93–4.91), who were of the female gender (OR = 1.75; 95% CI = 1.18–2.58), and who reported household impact from Typhoon Mangkhut (OR = 2.11; 95% CI = 1.08–4.12) were more likely to prepare for future typhoons (Table 5).

## 4. Discussion

The current study aims to explore the preparedness measures applied, the response activities conducted, and the impact experienced by urban residents when Typhoon Mangkhut landed in Hong Kong. The association among preparedness and response activities, impact, and willingness for future preparedness, along with their associating factors were also evaluated. More than 90.0% of respondents took up at least one preparedness measure against Typhoon Mangkhut, while more than 70.0% had applied at least one TSPM. People with a higher educational level, with a younger age, with a higher risk perception, and who practiced routine household preparedness were associated with a higher TSPM uptake. All respondents reported checking the weather information during the typhoon. The most commonly used information acquisition routes were television broadcast and mobile apps. However, during the typhoon, around 16.0% of respondents went out, and about three quarters of them were out for nonemergency or non-occupational reasons. About one-third of respondents reported some form of impact, and about 11% reported specific household impacts from Typhoon Mangkhut. Among those who had applied at least one TSPM, 12.9% reported household impacts. This study showed a higher risk perception was associated with more willingness to practice TSPM. Practicing TSPM was, however, not associated with the risk of household impacts.

### 4.1. Preparedness

Consistent with previous studies [18], this study found that a higher educational level, a higher risk perception, more routine emergency preparedness, and previous experience of impact were positively associated with people who engage in disaster preparedness. Studies from China [6], Japan [19], and the Philippines [20] have also reported positive links between risk perception and willingness to mitigate typhoon-related risks. The study from the Philippines showed experiencing previous impacts from typhoon disasters and a higher educational level was associated with better perception of preparedness against typhoons [20], similar to the associations found in Japan [19]. This current study also found people who engaged certain routine preparedness measures, such as having a first-aid kit and backup light source, were 1–3 times more likely to prepare for Typhoon Mangkhut. Studies about hurricanes in the United States did not find sociodemographic factors, such as education and age, as associating factors of having disaster supplies (e.g., food supplies, a first-aid kit, and a backup light source) [21]. This reflects the consistency in emergency preparedness for general disaster risk and disaster-specific risk in the community, which may be reinforced by the higher awareness and risk perception among this group.

Of note, compared to a study in Hong Kong (2012), the proportion of routine household preparedness has decreased in five years from 75.1% to 70.6% (practicing at least three out of five measures: having a first-aid kit, basic aid supplies, emergency food and drinking water, basic medication, and fire extinguishing equipment) [9]. Although the pattern difference was not of statistical significance (comparison of proportions: *p* = 0.06), the decrease suggested disaster risk awareness should be reinforced in the community for enhancing public health emergency preparedness with the face of more challenging meteorological conditions associated with climate change [22]. Inconsistent with another study which found 69.0% of people did not prepare during a severe weather warning, this study reported only 6.1% of respondents did not do any preparedness measures for Typhoon Mangkhut [10]. Besides the different sample methods adopted, the contrasting results may be due to a preference of Hong Kong residents to prepare for typhoons over other extreme weather events such as rainstorms, landslides, and thunderstorms, all of which are also categorized under severe weather warnings.

### 4.2. During the Typhoon

In spite of the Hong Kong Government’s Weather Services warning to stay indoors until winds were reduced to moderate severity, 16.0% of respondents had reported to have ventured outside when the storm was the strongest and most of them were out for nonemergency reasons. Although this figure is much lower in comparison to a previous local study (where 20.6% of respondents said that they would be “staying in a safe place until the T8 Tropical Cyclone Warning had passed” [12]), it may indicate the community regarded the Typhoon Mangkhut more seriously than previous typhoons. More efforts should be invested to prevent people from venturing outside for nonessential and unnecessary reasons. Leaving or travelling between shelters during strong winds may result in higher risks of injuries due to an increase length of exposure to hazards, e.g., flying objects [23]. This could also add an extra burden to the emergency response system and may even delay rescue for people with more pertinent needs.

Compared to other Chinese community [6], our urban community was more attentive to typhoon signals (100% vs. 54.8% in Zhejiang). Television was the most commonly used channel of communication for weather information in the community, which was on par with previous studies on information acquisition in Hong Kong about temperature events, infectious disease, and other disasters [12,24,25,26], in rural Zhejiang, China [6] and in the Philippines [20]. Despite the increase in use of mobile devices and apps, television still remains as the most commonly used channel for typhoon signals in both urban and rural communities.

### 4.3. Health Impacts

Official report indicated that no deaths and 450 injuries were directly resulted from the 2018 Typhoon Mangkhut in Hong Kong, yet this study found an injury rate of 0.77% among all respondents. If the event-specific injury rate was applied to the population of 7 million in Hong Kong (Table 2), there might be more than 50,000 unreported injury cases of varying severity. Although this is a crude estimation, the potential health impacts and the possible allocation of resources involved should be noted and considered for future disaster action plans. Most literature report injury figures based on public hospital records which may not include cases that did not seek professional medical assistance; the types of injury and other related disaggregate information also tend to be unavailable. Typhoon Hato in 2017, also categorized as a T10, killed at least ten people and caused 240 injuries in Macao [27]. In the west coastal cities of Japan, including Osaka and Kyoto, at least 11 deaths and 400 injuries have been reported due to Jebi in 2018, the strongest storm in Japan for 25 years [28], and in New York City, 43 deaths were reported caused by hurricane Sandy in 2012 [23]. The results of this study infer the injury figures reported in Macao [27], Japan [28] and New York City [23] might be underestimated as less severe cases which did not required hospitalizations though could cause public health problems might not be reported.

Currently, there are not many studies reporting the short-term health impacts of climate change-related storms on urban cities, especially for those with densely packed high-rise buildings like Hong Kong. This is important as the disaster impacts faced by the cities with and without high-rise buildings can vary drastically. For example, drowning due to flooding is one of the major health risks during storms [23], but it is less commonly reported in cities with densely packed high-rise buildings. A study found climate change has increased the intensity of typhoons in the East and Southeast Asia in the past few decades [22]. Coastal Asian cities in Korea, Japan, China, and Taiwan will become more vulnerable [22]. Coastal megacities such as Tokyo, Osaka, and Shanghai will be under an even higher risk due to the large and highly dense population. This study showed that injuries, mainly caused by falls, were the major health risks during the strong typhoon in Hong Kong. More evidence about the specific health risks during storms for high-rise building cities is needed.

### 4.4. What is the Gap Found in this Study

The biggest gap identified in this study is the effectiveness of the study TSPM in reducing storm-related impacts. This level of household preparedness may not be enough or appropriate for super typhoons as those who applied at least one TSPM were not associated with a lower risk of household impacts during the typhoon. This goes against the common understanding that appropriate preparedness should be able to reduce adverse effects during unfavourable events. Most of the household impacts reported, such as household damages and the suspension of the electricity or water supply, were uncommon typhoon impacts experienced in Hong Kong and are hardly preventable through applying the household protective measures mentioned in this study, such as taping windows. Other severe impacts were also reported from local media such as dizziness from swaying high-rise buildings, injuries from falling air conditioners, and the battering of construction sites where an elevator shaft had collapsed [29,30]. This further questions whether the current typhoon preparedness measures provide enough protection for super typhoons like Typhoon Mangkhut. Furthermore, there were disaster response and recovery measures which could not be addressed by individuals. One of the most common issues is road blocks, which in extreme events directly hinders relief efforts [31,32], but less is known about how it affects the daily routine of local residents, also referred to as secondary stressors [33]. The Hong Kong government reported 46,500 fallen trees that blocked major roads and several overhead rail lines [16]. Other unexpected health risks that arose included falling glass from twenty-story-high windows, of which mitigation efforts require more than just from homeowners but also input of various expertise from architecture and civil engineering.

### 4.5. Limitations

The cross-sectional nature of this study meant the inferences are limited to associations and the causation cannot be attributed. In addition, the landline telephone survey study design naturally excluded households without a landline; and in Hong Kong, residential landline penetration reported a decreasing rate from 102.6% in 2013 to 89.0% in 2018 [34]. Moreover, reporting bias may occur as as preparedness activities were self-reported and could not be validated. The current study also lacked the household location precision in Hong Kong and could not ascertain whether they were objectively at greater risk to assess the accuracy of the self-reported risk perceptions. The greater proportion of higher educated and higher income respondents might have also biased the results. Also, risk perceptions may have been biased based on the exposure to Typhoon Mangkhut, which may overestimate the association between typhoon exposure and risk perceptions. This study also only focused on the short-term impacts of the typhoon; the long-term impacts, such as psychiatric morbidity [35], and economic impact from the typhoon could not be included. Yet, despite the limitations, telephone surveys still offer the best opportunity to obtain quick overviews of various health and acute emergency impact status of the community [9,36]. As the survey was conducted just over two weeks following Typhoon Mangkhut, it may also reduced recall bias. 

### 4.6. Implications

With climate change, more frequent and more intense typhoons may affect the Southeast Asian regions [22,37]. Residents, as well as the government and other stakeholders, should be aware of the potential risks arising from climate change-related disasters and should be prepared for new challenges. Effective protective measures should also be identified, evaluated, and applied to minimize avoidable risk, especially for densely populated high-rise building cities. More proactive Health-EDRM measures, such as having windows checked and repaired before typhoon seasons, should be considered and promoted. Our results indicated that the practice of routine emergency preparedness, risk perception, and education level were associated with a higher uptake rate of protective measures against typhoons. This suggests increasing the awareness and educational promotion, in particular targeting people with a lower educational level. Relevant stakeholders should also help encourage community preparedness and evidence for future policy-level disaster preparedness plans [11]. Research with long-term follow up on trends and community perception and behaviour changes will be important to ensure relevant, appropriate, and effective household protective measures against future extreme weather events.

## 5. Conclusions

The results of this cross-sectional telephone survey study suggested that even the high uptake of TSPM might not be effective in preventing related adverse impacts in the face of a super typhoon. Effective protective measures should be identified, evaluated, and applied to cope with the possibility of more intense typhoons and other unprecedented risks. Low urban risk perception and the fact that a considerable proportion of respondents venturing out during strong winds found in this study further urges the need to raise the awareness and preparedness practices among the community against typhoons and strong wind events. Education level and risk perception were found associated with the uptake of the TSPM; hence, educational promotion is suggested to raise awareness and to encourage the community to be better prepared. This is of timely importance since climate-related natural disasters are expected to increase in frequency and severity due to the effects of climate change [22]. The commonplace and prevalence of disasters, no matter the scale, should not inhibit emergency and disaster contingency plans from being updated or further adapted by the latest scientific evidence.

## Figures and Tables

**Table 1:** The uptake rate of preparedness activities applied on usual days and for Typhoon Mangkhut.

Types of Household Preparedness Measures (*n* = 521)	Health-EDRM Implications	Routine Emergency Preparedness	Typhoon Mangkhut Preparedness
**General emergency preparedness measures**			
Food Supply	To ensure food security and to maintain proper nutritional intake	432 (82.9%)	355 (68.1%)
Drinking water	To have clean water for sanitation, hydration, and food preparation	255 (48.9%)	197 (37.8%)
Basic medication (e.g., pain relievers)	To deal with acute clinical symptoms related to pains and fever	488 (93.7%)	242 (46.4%)
Long term medication (2 weeks)	To sustain treatment plan(s) and the continuous management of chronic diseases	279 (53.6%)	148 (28.4%)
Backup light source	To provide visual aid to prevent injuries such as falling	417 (80.0%)	281 (53.9%)
Backup electrical source	To elongate the functionality of electronical appliances such as medical equipment or cooking apparatuses	109 (20.9%)	98 (18.8%)
First-aid kit	For the immediate treatment and mitigation of emergencies and accidents	288 (55.3%)	-
Basic first-aid supplies, e.g., Band-Aids and ace bandages	For the treatment and mitigation of minor injuries	496 (95.2%)	-
Fire extinguishing equipment	To control the fire hazard and to prevent fire-related injuries	63 (12.1%)	-
**Typhoon-specific preparedness measures (TSPM)**			
Taped windows	To reduce shattered glass pieces for injury prevention	-	268 (51.4%)
Collect or tied down items that can be blown away (e.g., flower pots)	To reduce the risk of blunt force trauma from objects carried by the storm	-	273 (52.4%)
Anti-flooding, leaking, and seeping measures	To reduce injury risks related to slippery surfaces and allergies or airborne toxins related to mould and fungi	-	195 (37.4%)

Health-EDRM: Health-Emergency and Disaster Risk Management.

**Table 2:** The descriptive statistics about demographics, perception, preparedness, and impact.

Demographics	Sampled Respondents (*n* = 521)		HK 2016 Population by Census Data (*n* = 6,506,130)		Sample vs. Census *p*-Value ^a^
	*n*	%	*n*	%	
Gender					
Male	221	42.4%	2,947,073	45.3%	0.202 ^b^
Female	300	57.6%	3,559,057	54.7%	
Age					
18–24	63	12.1%	785,981	12.1%	0.005 *
25–44	154	29.6%	2,228,566	34.3%	
45–64	224	41.5%	2,328,430	35.8%	
≥65	80	15.4%	1,163,153	17.9%	
Area of residence					
Hong Kong Island	102	19.6%	1,120,143	17.2%	0.219
Kowloon	164	31.5%	1,987,380	30.6%	
New Territories	254	48.8%	3,397,499	52.2%	
Education attainment					
Primary and below	56	10.7%	1,673,431	25.7%	<0.001 *
Secondary	195	37.4%	2,841,510	43.7%	
Post-secondary	265	50.9%	1,991,189	30.6%	
Marital status					
Single	212	40.7%	2,708,709	41.6%	0.695 ^b^
Married	309	59.3%	3,797,421	58.4%	
Income					
<2000–9999	45	9.4%	480,117	19.2%	<0.001 *
10,000–19,999	73	15.2%	547,784	21.8%	
20,000–39,999	160	33.4%	699,450	27.8%	
≥40,000	201	42.0%	782,383	31.2%	
Perceived home to be at high risk during typhoons (*n* = 520)					
Yes	49	9.4%	-	-	-
No	471	90.4%	-	-	-
Impact from Typhoon Mangkhut (*n* = 521)					
Yes	174	33.4%	-	-	-
No	347	66.6%	-	-	-
Practiced at least 1 typhoon-specific preparedness (*n* = 521)					
Yes	387	74.3%	-	-	-
No	134	25.7%	-	-	-
Went out when typhoon signal was T8 or above (*n* = 520)					
Yes	83	16.0%	-	-	-
No	437	84.0%	-	-	-

^a^ The *χ^2^* test was used to measure the overall difference between this survey and the 2016 Hong Kong Population Census data. A *p*-value < 0.05 indicates a significant difference. ^b^ The *χ^2^* test with continuity correction was used. * *p* < 0.05.

**Table 3:** The association of demographics with practiced typhoon-specific preparedness measures (TSPM) and risk perception.

Characteristics		Practiced at Least 1 Typhoon-Specific Preparedness Measure (TSPM) ^				
		*χ^2^* Test; *n* = 521 *			Logistic Regression; *n* = 515	
		Yes	No	*p*-Value	OR (95% CI)	*p*-Value
Gender	Male	161 (41.6%)	60 (44.8%)	0.522	1	
	Female	226 (58.4%)	74 (55.2%)		1.16 (0.76–1.77)	0.493
Age	18–24	50 (12.9%)	13 (9.7%)	<0.001	1.75 (0.75–4.07)	0.193
	25–44	131 (33.9%)	23 (17.2%)		2.80 (1.39–5.65)	0.004
	45–64	158 (40.8%)	66 (49.3%)		1.43 (0.81–2.53)	0.219
	≥65	48 (12.4%)	32 (23.9%)		1	
Education attainment (*n* = 516)	Primary and below	37 (9.6%)	19 (14.4%)	<0.001	0.59 (0.29–1.22)	0.156
	Secondary	127 (33.1%)	68 (51.5%)		0.48 (0.30–0.77)	0.003
	Post-secondary	220 (57.3%)	45 (34.1%)		1	
Income (*n* = 479)	<2000–9999	27 (7.6%)	18 (14.8%)	0.110	-	-
	10,000–19,999	55 (15.4%)	18 (14.8%)		-	-
	20,000–39,999	119 (33.3%)	41 (33.6%)		-	-
	≥40,000	156 (43.7%)	45 (36.9%)		-	-
Perceived home to be at high risk during typhoons (*n* = 520)	Yes	43 (11.1%)	6 (4.5%)	0.023	2.63 (1.07–6.50)	0.036
	No	343 (88.9%)	128 (95.5%)		1	

^ Retrieved/stored outdoor items that could be blown away, applied anti-leaking or anti-seeping measures, or taped windows. * The sample size was 521 unless stated otherwise due to missing data.

**Table 4:** The associating factors of typhoon household impact.

Characteristics		Household Impact due to Typhoon Mangkhut				
		*χ^2^* Test; *n* = 521 *			Logistic Regression; *n* = 515	
		Yes	No	*p*-Value	OR (95% CI)	*p*-Value
Gender	Male	19 (32.8%)	202 (43.6%)	0.114	-	-
	Female	39 (67.2)	261 (56.4%)		-	-
Age	18–24	8 (13.8%)	55 (11.9%)	0.132	-	-
	25–44	23 (39.7%)	131 (28.3%)		-	-
	45–64	23 (39.7%)	201 (43.4%)		-	-
	≥65	4 (6.9%)	76 (16.4%)		-	-
Education attainment (*n* = 516)	Primary and below	3 (5.5%)	53 (11.5%)	0.184	-	-
	Secondary	18 (32.7%)	177 (38.4%)		-	-
	Post-secondary	34 (61.8%)	231 (50.1%)		-	-
Income (*n* = 479)	<2000–9999	3 (5.8%)	42 (9.8%)	0.533	-	-
	10,000–19,999	6 (11.5%)	67 (15.7%)		-	-
	20,000–39,999	21 (40.4%)	139 (32.6%)		-	-
	≥40,000	22 (42.3%)	179 (41.9%)		-	-
Floor levels	<6	18 (31.6%)	91 (19.7%)	0.185	-	-
	6–15	20 (35.1%)	166 (36.0%)		-	-
	16–25	11 (19.3%)	113 (24.5%)		-	-
	≥26	8 (14.0%)	91 (19.7%)		-	-
Perceived home to be at high risk during typhoons	Yes	17 (29.3%)	32 (6.9%)	<0.001	5.16 (2.63–10.14)	<0.001
	No	41 (70.7%)	430 (93.1%)		1	
Practiced at least one typhoon specific preparedness measure	Yes	50 (86.2%)	337 (72.8%)	0.027	2.02 (0.92–4.45)	0.080
	No	8 (13.8%)	126 (27.2%)		1	

* The sample size was 521 unless stated otherwise due to missing data.

**Table 5:** The multivariable logistic regression for willingness to prepare for future typhoons.

Characteristics		Willingness to Practice Future Preparedness for Typhoons				
		*χ^2^* test; *n* = 521 *			Logistic Regression; *n* = 515	
		Yes	No	*p*-Value	OR (95% CI)	*p*-Value
Gender	Male	94 (36.2%)	127 (48.7%)	0.004	1	
	Female	166 (63.8%)	134 (51.3%)		1.75 (1.18–2.58)	0.005
Age	18–24	33 (12.7%)	30 (11.5%)	0.116	-	-
	25–44	88 (33.8%)	66 (25.3%)		-	-
	45–64	100 (38.5%)	124 (47.5%)		-	-
	≥65	39 (15.0%)	41 (15.7%)		-	-
Education attainment (*n* = 516)	Primary and below	31 (12.1%)	25 (9.7%)	0.057	1.32 (0.63–2.77)	0.471
	Secondary	84 (32.7%)	111 (42.9%)		0.65 (0.41–1.03)	0.064
	Post-secondary	142 (55.3%)	123 (47.5%)		1	
Income (*n* = 479)	<2000–9999	22 (9.0%)	23 (9.8%)	0.096	1.36 (0.64–2.88)	0.428
	10,000–19,999	47 (19.3%)	26 (11.1%)		2.29 (1.21–4.34)	0.011
	20,000–39,999	76 (31.1%)	84 (35.7%)		1.03 (0.65–1.64)	0.891
	≥40,000	99 (40.6%)	102 (43.4%)		1	
Perceived home to be at high risk during typhoons	Yes	33 (12.7%)	16 (6.2%)	0.011	1.43 (0.71–2.90)	0.319
	No	227 (87.3%)	244 (93.8%)		1	
Practiced at least one typhoon specific preparedness measure	Yes	222 (85.4%)	165 (63.2%)	<0.001	3.07 (1.93–4.91)	<0.001
	No	38 (14.6%)	96 (36.8%)		1	
Household impacted by Typhoon Mangkhut	Yes	40 (15.4%)	18 (6.9%)	0.002	2.11 (1.08–4.12)	0.028
	No	220 (84.6%)	243 (93.1%)		1	

* The sample size was 521 unless stated otherwise due to missing data.

## Appendix A

**Table A1:** Association of practicing at least 1 typhoon-specific preparedness measure (TSPM) and routine household preparedness measures.

Multivariable Logistic Regression—Practiced at Least 1 Typhoon-Specific Preparedness (*n* = 515)					
Variables					
Routine Household Preparedness	First-Aid Kit	Food Supply	Basic Medicine	Fire Extinguishing Equipment	Back Up Light Source
Yes	1.79 (1.18–2.73) **	1.80 (1.08–3.03) *	1.69 (0.75–3.65)	3.13 (1.36–7.20) **	1.69 (1.03–2.77) *
No	1	1	1	1	1
**Education**					
Primary	0.65 (0.31–1.35)	0.65 (0.31–1.35)	0.62 (0.30–1.29)	0.62 (0.30–1.28)	0.58 (0.28–1.19)
Secondary	0.51 (0.31–0.82) **	0.48 (0.30–0.78) **	0.48 (0.30–0.78) **	0.47 (0.29–0.77) **	0.49 (0.30–0.79) **
Post-secondary	1	1	1	1	1
**Gender**					
Male	1	1	1	1	1
Female	1.14 (0.75–1.75)	1.11 (0.72–1.71)	1.16 (0.76–1.78)	1.20 (0.78–1.85)	1.16 (0.76–1.78)
**Age**					
18–24	1.60 (0.69–3.75)	1.63 (0.70–3.80)	1.70 (0.73–3.97)	1.73 (0.74–4.04)	1.70 (0.73–3.96)
25–44	2.61 (1.28–5.30) **	2.69 (1.33–5.46) **	2.69 (1.33–5.46) **	2.79 (1.38–5.66) **	2.89 (1.43–5.87) **
45–64	1.33 (0.75–2.37)	1.43 (0.81–2.54)	1.40 (0.79–2.48)	1.37 (0.77–2.43)	1.49 (0.84–2.64)
65+	1	1	1	1	1
**Perceived home to be at high risk during typhoons**					
Yes	2.67 (1.08–6.60) **	2.82 (1.14–7.00) *	2.65 (1.08–6.55) *	2.65 (1.07–6.55) *	2.49 (1.01–6.15) *
No	1	1	1	1	1

* *p* < 0.05. ** *p* < 0.01.
